# Highlights on Recent Developments of Heterogeneous and Homogeneous Photocatalysis

**DOI:** 10.3390/molecules26010023

**Published:** 2020-12-23

**Authors:** Francesco Parrino, Giovanni Palmisano

**Affiliations:** 1 Department of Industrial Engineering, University of Trento, Via Sommarive 9, 38123 Trento, Italy; francesco.parrino@unitn.it; 2 Department of Chemical Engineering, Khalifa University of Science and Technology, P.O. Box 127788, Abu Dhabi, UAE; 3 Research and Innovation on CO_2_ and H_2_ (RICH) Center, Khalifa University of Science and Technology, P.O. Box 127788, Abu Dhabi, UAE

Photocatalysis emerged in the last decades as a versatile technology, whose applications range from environmental remediation to hydrogen production, energy harvesting, and organic synthesis, with exciting examples also in medicine, electronics, and advanced functional materials. While homogeneous photocatalysis has a glorious and consolidated tradition, especially in the field of chemical synthesis, heterogeneous photocatalysis came into the limelight more recently, due to the appealing advantages provided by processes occurring at solid–fluid interfaces. Even if fundamental research should be never abandoned, because it is essential nourishment for applied research, photocatalysis is mature enough to demonstrate its industrial feasibility. This change in direction is especially required as far as environmental remediation is concerned, where up to now the technology transfer from lab to industry is probably the bottle neck of several processes. In this sense, we believe that novel photocatalytic materials could be defined as promising for environmental remediation only if high photocatalytic activity (possibly in the visible region) is accompanied by the required robustness, reusability, and reliability that justify industrial investments. At the same time, engineering and reactor design issues are of the utmost importance to enable scale-up of the processes and require further research. This scenario is reflected in the topics approached in the photocatalysis papers recently published in the journal Molecules. In fact, as detailed below, the majority of them deal with bare or diversely modified TiO_2_, which, more than any other semiconductor, combines high photoactivity for both degradation and disinfection, robustness, abundance, and low cost.

Non-metal doping has been investigated with the aim of extending the absorption ability of TiO_2_ toward the visible range. Carbon and nitrogen co-doping have been presented by Janus et al. [1] as an efficient approach to extend the photocatalytic activity toward the visible range. The materials have been embedded in concrete plates and provided total inactivation of Escherichia coli under simulated solar light irradiation. Pablos et al. [2] studied the Escherichia coli inactivation under UV-vis light by means of electrochemically assisted photocatalysis, by using nitrogen and fluorine co-doped TiO_2_ materials. Gurkan et al. [3] investigated the structural and opto-electronic features of selenium and nitrogen co-doped TiO_2_ and tested the materials in the photocatalytic degradation of a target pollutant (4-nitrophenol) in water. Coupling TiO_2_ with other semiconductors active in the visible range is another expedient to enhance the light responsive features of TiO_2_. For instance, Nevarez-Martinez at al. [4] synthetized nanotube arrays of TiO_2_-MnO_2_ by electrochemical anodization, and used them for the photocatalytic degradation of toluene in gas phase under visible light irradiation. Hong et al. [5] produced recyclable TiO_2_/Fe_2_O_3_ nanocomposites from ilmenite and applied these materials for the degradation of Rhodamine B under visible and solar light irradiation. A review has been published on the role of TiO_2_ coupled with carbon materials, such as carbon nanotubes, graphene, and carbon quantum dots, in the photocatalytic degradation of three target pharmaceutical compounds [6]. Aside from TiO_2_, some investigations on other photoactive materials and their environmental applications have been published, even if fewer in number with respect to those on TiO_2_.

As abovementioned, research on scale up issues and reactor design aimed at promoting industrial exploitation of photocatalytic reactions is still required. Applications for the purification of air already found benign market acceptance and are a step forward with respect to those in liquid phase. Accordingly, Molecules published the contribution by Alfano et al. [7], reporting a methodology for the integrated design of photocatalytic reactors for air cleaning purposes from the development of intrinsic kinetic models, ultimately targeting reactor scale-up and optimization. Dumont et al. [8] described theoretical and experimental approaches for the determination of the Clean Air Delivery Rate (CADR) of photocatalytic air purifiers. Different reactor designs have been proposed in other contributions. For instance, Montalvo et al. [9] proposed a semi-pilot rotating photocatalytic reactor, and Pellegrino et al. [10] described the photocatalytic activity of a cordierite-honeycomb-supported TiO_2_ film in a liquid–solid photoreactor for water treatments applications.

It is worth mentioning that the combination of heterogeneous photocatalysis with other advanced oxidation processes or physical methods [11,12] is a viable and competitive tool to boost industrial appeal. In fact, synergetic effects could reduce the drawbacks of the contributing single technologies, simultaneously increasing the efficiency of the whole process. To this aim, Beltran and Rey [13] published a review in Molecules on solar or visible light photocatalytic ozonation by discussing on the effects of the main intervening parameters.

A completely different situation holds when photocatalysis is used for the synthesis of high-value-added compounds as an alternative to traditional synthetic paths. Homogeneous photocatalysis is a well-established strategy for organic synthesis, as confirmed by the fact that the majority of the papers published in Molecules on photocatalytic synthetic processes occur in the presence of organometallic complexes in a homogeneous phase. Protti et al. [14] exploited the acidity of photo-excited molecules to drive C–C and C–S bond formation processes by providing a viable alternative to the use of aggressive acids and harsh conditions. Iron-based organometallic complexes have been used under visible light irradiation to reduce CO_2_ into CO [15], ruthenium complexes bearing pyridine–quinoline or terpyridine ligands were utilized in the atom transfer radical addition of haloalkanes to olefins [16], while the effects of some reaction parameters were investigated for the photodimerization of 2-anthracenecarboxylate catalyzed by platinum (II) complexes [17]. A comprehensive review on photocatalytic difluoromethylation reactions of aromatic compounds and aliphatic multiple C–C bonds in the presence of organometallic compounds have been also published [18].

On the other hand, heterogeneous photocatalysis for synthetic purposes is less represented. In fact, only one paper has been published on the selective partial oxidation of cyclohexane [19]. This fact reflects a general mistrust with respect to the potentiality of heterogeneous photocatalysis for the synthesis of relevant industrial compounds. Production of raw materials [20], naturally occurring compounds [21], or valuable intermediates [22] in good to excellent yields have been accomplished, but correspondent advancements in the engineering of the processes are still required. Moreover, some of the contributions only report chromatographic or spectroscopic evidence of the formed products, without any concern on purification and separation, at least on a gram scale. In our opinion, advancements in membrane reactors [23] could support the development of this intriguing field, which offers competitive alternatives to classical synthetic protocols under milder conditions and in fulfilment of the green chemistry principles.
